# Sulphate of Quinine in Fever

**Published:** 1849-04

**Authors:** 


*Sulphate of Quinine in Fever.*—I repeat, if you give quinine when the pulse is at one hundred and thirty beats to the minute, and falling, (premising of course the necessary depletions,) and give it in sufficient quantity, say five grains, with a little ipecac. every hour, that you will, in a very large majority of cases, find that the pulse will fall rapidly under its influence, until by the time the fifth portion is exhibited, your patient is altogether free from fever. Whereas, if you give it when the pulse is at eighty beats to the minute, and rising, you will invariably increase the action of the heart in force and frequency, and if pushed to any considerable extent, cause the death of your patient. It is also well worthy of remembrance, that quinine given at an improper time, is not only calculated to excite fever, and do an immense amount of injury in that way, but oft repeated observations have confirmed me in the opinion, long since entertained, that it materially lessens the capability of that article to impress the system favorably afterwards; i. e. after you have succeeded in effecting a remission or intermission of the fever; thus depriving as of the use, to a certain extent, of the most valuable agent known to medicine in combatting this dangerous malady.

I will now attempt to give you some idea of our treatment of the so-called congestive fevers of our country, in the cold stage. The after treatment, you will be able to learn much better than I can teach, in the schools, from the books and by your own observation. Indeed, most of the writers describe this part of the treatment with sufficient accuracy, but I think they fail to point out with clearness, the *extent* necessaray to carry the means that are to be used, and it is to this that I would call your particular attention. You will bear in mind that there is great danger of death in the first or cold stage, in the malignant cases; re-action never taking place unless nature is assisted by art. So insidious are these attacks sometimes, that the patient is not aware of it himself for sometime after it commences. The legs and arms gradually become cold, the coldness commencing at the free extremity of the limb, and gradually extending up to the body. He is at last reminded of his condition by a sense of great weight or oppression about the epigastrium, which continues to deepen until he is utterly insensible to all surrounding objects. Sometimes I have seen patients with all the evidences of deep congestion, without the intellectual faculties being at all impaired—they complain of no particular pain, but of an indefinable sense of misery. I will not stop to theorize upon the probable cause of these malignant diseases, for of this yon will find more than enough already written. It is doubtless the introduction of some subtle poison into the system, causing deep lesion of the nervous centers, affecting thereby calorification, and preventing the heart from sending the blood to the extreme capillaries. Differences in age, sex, constitution, idiosyncrasy, &c., will cause differences in the phenomena presented in different cases, but the indications of treatment are nearly the same in all. They are to warm the extremities, equalize the circulation, relieve the congestions and inflammation, allay nervous irritability, and put a stop to the periodic tendency of the disease. To accomplish which, external heat, cupping over the epigastric and right and left hypochondriac regions and along the spine, warm fomentations, the application of sinapisms to the extremities, spine, chest and abdomen, promoting the secretions, and to fill the two last indications, to introduce a sufficient quantity of the antiperiodic agent and morphine: all these may be brought into requisition, and to the *extent justified by the urgency of the symptoms.* When called to treat one of these cases, I direct eight to ten gallons of water to be heated, ears of boiled corn, bricks heated; and a pint of spirits of turpentine, with an equal quantity of vinegar, to be put into a vessel and made boiling hot, into which put half pound of mustard and the same quantity of cayenne pepper. With this strips of flannel are to be saturated, and the extremities enveloped from the fingers and toes to the trunk, and then the heated mixture to be poured on them. A small blanket torn into strips of the proper width answer the best purpose for encircling the limbs. The hot bricks should then be put about the feet and legs, and the hot corn about the body. Previously, however, to doing this, the patient should be cupped over the liver and stomach, and the warm fomentations used; also, cupped along the spine, and the sinapisms applied along the spine from the occiput to the sacrum, and over the lower part of the chest and abdomen.

The most important inquiry now is, at what time, and in what quantity, shall quinine be given? This will depend upon circumstances. If we have reason to believe that it is a congestive ague of the quotidian type, we need be in no great hurry about administering quinine; but as we cannot always arrive at a correct conclusion upon this point, it will be safest to commence giving it as soon as there is a perfect intermission. But if it be congestive remittent fever, I hold that a perfect re-action should *first* be established, and then at the first appearance of a decline of the pulse, the quinine should be commenced. About five grains every hour for five or six times is the quantity that I usually give; and eight grains, I think, is the largest amount that I have ever given at a dose in these cases.

In conclusion, I will say that it would be extremely difficult to enumerate all the cases of disease, and conditions of body that may be benefitted by the use of quinine. But, regarding it as a pure tonic in small doses, and as *the* great anti-periodic agent, I should say that its use was indicated in nearly all cases requiring a tonic treatment, alternating or combining it with other tonics; and in all cases of disease that commence with, or assume during their course, the periodic character. Of these latter, you will find a vast many in the South; for, in the language of some of the ancient writers, intermittent fever, at times, seems to have the power of forcing all other diseases to put on its livery.—*Med. Examiner.*

